# Reproducibility of Mesopic and Photopic Pupil Sizes in Myopic Children Using a Dedicated Pupillometer with Human-Assisted or Automated Reading

**DOI:** 10.3390/jpm13020273

**Published:** 2023-01-31

**Authors:** Anders Hvid-Hansen, Per Bækgaard, Nina Jacobsen, Jesper Hjortdal, Flemming Møller, Line Kessel

**Affiliations:** 1 Department of Ophthalmology, Copenhagen University Hospital—Rigshospitalet-Glostrup, DK-2600 Glostrup, Denmark; 2 Department of Applied Mathematics and Computer Science, Technical University of Denmark, DK-2800 Kongens Lyngby, Denmark; 3 Department of Clinical Medicine, University of Copenhagen, DK-2200 København N, Denmark; 4 Department of Ophthalmology, Aarhus University Hospital, DK-8200 Aarhus N, Denmark; 5 Department of Ophthalmology, University Hospital of Southern Denmark—Vejle Hospital, DK-7100 Vejle, Denmark

**Keywords:** pupillometry, myopia, reproducibility, limits of agreement, automated reading

## Abstract

This study aimed to investigate the reproducibility of pupil size measurements over time and between reading methods when comparing human-assisted reading to automated reading. Pupillary data were analyzed on a subset of myopic children enrolled in a multicenter randomized clinical trial on myopia control with low-dose atropine. Pupil size measurements were obtained prior to randomization at two time points (screening and baseline visits) using a dedicated pupillometer under mesopic and photopic conditions. A customized algorithm was built to perform automated readings, allowing comparisons between human-assisted and automated readings. Reproducibility analyses followed the principles of Bland and Altman and included the calculation of the mean difference between measurements and limits of agreement (LOA). We included 43 children. Mean (standard deviation) age was 9.8 (1.7) years and 25 (58%) children were girls. Using human-assisted readings, reproducibility over time showed mesopic mean difference of 0.02 mm with LOA from −0.87 mm to 0.91 mm, whereas photopic mean difference was −0.01 mm with LOA from −0.25 mm to 0.23 mm. Reproducibility between human-assisted and automated readings was also higher under photopic conditions, with mean difference of 0.03 mm and LOA from −0.03 mm to 0.10 mm at screening and mean difference of 0.03 mm and LOA from −0.06 mm to 0.12 mm at baseline. Using a dedicated pupillometer, we found that examinations performed under photopic conditions demonstrated higher reproducibility over time and between reading methods. We speculate whether mesopic measurements are sufficiently reproducible to be monitored over time. Furthermore, photopic measurements may be of greater relevance when evaluating the side effects of atropine treatment, such as photophobia.

## 1. Introduction

The pupil functions as an aperture and regulates the amount of light that reaches the retina. The pupil size varies between 2 and 8 mm, and the size is controlled by the autonomic nervous system [1]. The pupil constricts in response to light exposure [2,3] and near fixation [1,4], whereas cognitive engagement causes pupil dilation [5]. Refractive errors may also influence pupil size [3,6]. Pharmacologic agents may both dilate and constrict the pupil [4,7,8,9]. Atropine, a non-specific muscarinic receptor antagonist, causes pupil dilation and is currently the most effective therapy for controlling myopia progression in children and adolescents [10,11].

Myopia is among the most prevalent eye disorders globally, and importantly the prevalence is increasing worldwide. It has been estimated that half of the world’s population will be myopic by 2050 [12,13]. The search for interventions to control myopia progression is motivated by the increased risk of sight-threatening complications associated with myopia and particularly high myopia (−6 diopters (D) or more), including retinal detachment, cataracts, and myopic maculopathy [13,14,15]. Current strategies to reduce myopia progression include behavioral, optical, and pharmacological approaches [16].

Because atropine-induced pupil dilation can cause photophobia, pupillometry has been an important safety measure in randomized clinical trials investigating the myopia-controlling effect of atropine [4]. However, changes in minimum pupil size induced by different atropine dosages have varied greatly across studies [17,18,19], which has been ascribed to the use of different pupillometers and luminosity levels during examinations [4]. To date, only one study investigated the repeatability of minimum pupil size measurements in myopic children [4], whereas the reproducibility of these measurements over time has not been reported.

In this study, we use a dedicated pupillometer to investigate two aspects of reproducibility in minimum pupil size measurements: (1) the reproducibility of mesopic and photopic pupil sizes over time and (2) the reproducibility between reading methods when comparing human-assisted reading to automated reading of mesopic and photopic pupil size measurements.

## 2. Materials and Methods

### 2.1. Participants

We evaluated the pupillary measurements performed at the screening and baseline visits in myopic children enrolled in the Low-dose Atropine for the Prevention of Myopia Progression in Danish Children (APP) study. The APP study is an ongoing, randomized, double-masked, placebo-controlled, multicenter study designed to investigate the efficacy and safety of a 0.1% atropine loading dose and 0.01% atropine alone eye drops in halting myopic progression. Study sites included Copenhagen University Hospital, Aarhus University Hospital, and University Hospital of Southern Denmark.

Participants were recruited among subjects referred by ophthalmologists and optometrists. Additionally, self-referral by parents was accepted. We included children aged 6 to 12 years with myopia (spherical component by cycloplegic autorefraction in at least one eye) of ≤−1 D if age was ≥6 to <9 years, or ≤−2 D if age was ≥9 to ≤12 years, and astigmatism of less than −1.5 D. Exclusion criteria were ocular pathology (e.g., amblyopia, strabismus, keratoconus, retinal dystrophies, and previous eye surgery); systemic diseases (e.g., connective tissue disorders and severe cardiac or respiratory illness); developmental disorders and delays; previous myopia control with atropine, 7-methylxanthine, orthokeratology lenses, and/or other optical interventions; known allergy to trial medication; and non-compliance to eye examinations.

The present study included data from the Copenhagen University Hospital because pupillometry was performed both at screening and baseline visits exclusively at this site. The division between the screening and baseline visit originated from the intention to maximize future treatment adherence to daily eye drop administration by offering consenting and eligible participants a trial period of at-home lubricant eye drop administration. No other treatment was initiated between the two visits. At the baseline visit, participants were reassessed, randomized to trial medication, and scheduled for future follow-ups according to the study protocol.

### 2.2. Approvals

The study was approved by the Committee on Health Research Ethics for the Capital Region of Denmark (reference no.: H-18043987), the Danish Medicines Agency (reference no.: 2018-040088), and The Danish Data Protection Agency (reference no.: P-2022-85). GCP units at Copenhagen University Hospital, Aalborg and Aarhus University Hospitals, and Odense University Hospital monitored the study sites according to the GCP quality standards. The study was registered in the European Union Drug Regulating Authorities Clinical Trials Database (EudraCT: 2018-001286-16) and at www.clinicaltrials.gov (accessed on 2 January 2023) (NTC no.: NCT03911271) before initiation. The study followed the tenets of the Declaration of Helsinki. We obtained written informed consent from parents and verbal assent from the children.

### 2.3. Examinations

This study included demographic, biometric, and refractive data from study participants enrolled at the Copenhagen University Hospital. We obtained the age and sex of the participants from the Danish central person registry. Mesopic and photopic pupil sizes were measured during the screening and baseline visits using a computer-based automated binocular pupillometer (DP-2000 Human Laboratory Pupillometer, NeurOptics, Irvine, CA, USA). Measurements were performed in a darkened room with blackout curtains and room lights off. Both eyes were stimulated simultaneously with white light under mesopic (4 lux) and photopic (300 lux) conditions. Continuous binocular pupil size measurements were performed before (3 s) and during (10 s) light stimulus. A total of five minimal pupil size measurements (with a maximum tolerability range of 1.0 mm) were performed during light stimulus for each light condition, completing all mesopic measurements first. Human-assisted readings of pupil diameter were performed using a built-in graphical user interface (GUI) on the pupillometer (DesktopTracker, version 137, NeurOptics, Irvine, CA, USA). If the GUI reported invalid results due to blink artifacts or lost pupil tracking, the minimum pupil size under light stimulus was marked and entered manually in the GUI. Trained investigators performed pupil size measurements and subsequent readings. Each continuous binocular examination was saved as a data file, which was exported and underwent automated data reading using a customized algorithm, see *Automated data reading*. Axial length (AL) was measured using a swept-source optical coherence tomography (SS-OCT)-based biometer IOLMaster 700 (Carl Zeiss Meditec AG, Jena, Germany). Cycloplegic autorefraction was obtained using the Retinomax K-plus 3 (Right Mfg. Co. Ltd., Tokyo, Japan) 30 min after a complete cycloplegia regimen, consisting of at least two drops of cyclopentolate 1% (Minims Cyclopentolate Hydrochloride 1%, Bausch & Lomb Nordic AB, Stockholm, Sweden), which was administered to both eyes 5 min apart. Cycloplegic spherical equivalent (SE) was calculated as spherical power plus half cylinder power. Cycloplegic autorefraction was the only measurement performed under cycloplegia.

### 2.4. Automated Data Reading

For each measurement, the pupil size readings generated in the GUI and complete time series of binocular absolute pupil size, their corresponding timestamp, indicated validity by the built-in reading software, and stimuli light luminosity were extracted from the data files by a custom Python (version 3.7.13) script using packages Pandas (version 0.24.0) [20], NumPy (version 1.16.2) [21], and SciPy (version 1.2.1) [22].

For each pupil size time series and eye separately, a Hampel-based filter [23,24] over a window of 1.4 s was used to remove outliers more than 1.5 mm or more than t=3 times the median absolute deviation estimator [25]. All data points marked as invalid by the built-in reading software were also discarded, as were any data points less than 2 mm, larger than 9 mm, or more than 0.5 mm away from the previous data point, which would require constriction velocity higher than approximately 15 mm per second.

**Figure 1:**
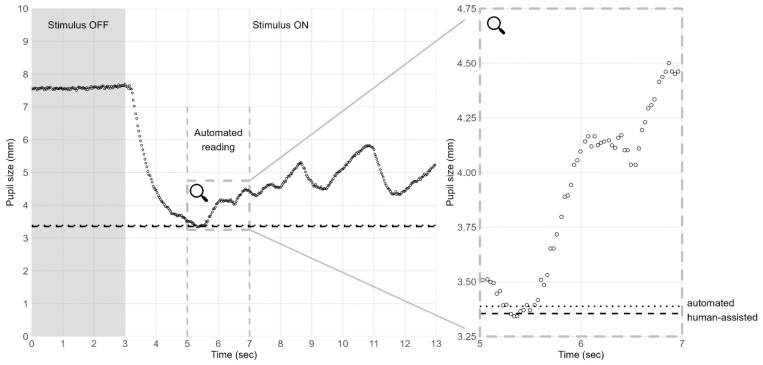
The inherent difference between human-assisted and automated readings. **Notes:** An example of continuous pupil size measurement over time under mesopic conditions. Pupil size is plotted during 3 s of darkness (Stimulus OFF) and 10 s of light (Stimulus ON). The pupil size decreases from about 7.5 mm to about 3.4 mm (3 to 5.5 s), escapes to larger size (5.5 to 8.5 s) and oscillates at about 5 mm (8.5 to 13 s). Vertical dashed lines indicate the 2-s interval of automated reading. Magnified insert highlights the inherent difference between reading methods as the automated reading (horizontal dotted line) was configured to report a 10% percentile, whereas the human-assisted reading (horizontal dashed line) allowed for reporting the minimum pupil size based on a single data point. **Abbreviations:** sec = second.

**Figure 2:**
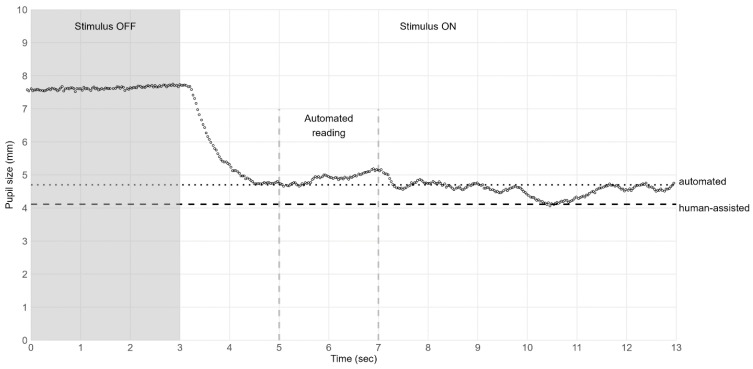
Difference between reading methods include the influence of latent pupillary constriction. **Notes:** An example of latent pupillary constriction resulting in a deviation between reading methods. Pupil size is continuously recorded during 3 s of darkness (Stimulus OFF) and 10 s of light (Stimulus ON). The pupil constricts from about 7.7 mm to about 4.7 mm (3 to 5.5 s), escapes to larger size (5.5 to 7 s), and latently constricts again (primarily at 10 to 11 s) to about 4.1 mm. Vertical dashed lines indicate the 2-s interval of automated reading. Automated (horizontal dotted line) and human-assisted (horizontal dashed line) readings report minimum pupil sizes of 4.7 mm and 4.1 mm, respectively. As illustrated, the latent constriction around 10.5 s is only registered by the human-assisted reading, thus contributing to the bias between reading methods. The observed latent constriction could support the idea that mesopic conditions allow more pupil-regulatory impact from near fixation and cognitive activity during measurement. **Abbreviations:** sec = second.

As for the remaining data points, the median and 10% percentile were calculated in the light interval between 2 and 4 s after light stimulus onset ($p_{light,median},$ $p_{light,10\%}$). We selected this interval from a heuristic approach, balancing the capture of maximum constriction with the exclusion of pupillary escape and latent pupillary constrictions (Figure 1 and Figure 2). A quality score $q\in \left[0,\;1\right]$ was assigned to each calculation based on the ratio of non-discarded data points to all data points in the interval, and a weighted score was assigned as $w=q\times e^{-2\times d}$, where d was the absolute difference between the median and the 10% percentile.

For each light condition, all 10% percentiles from light intervals with q>0.5 were used to calculate a robust weighted mean of the possible five measurements, $p_{i,light,10\%}$, as an estimator of the filtered minimal pupil size during light stimuli, $\bar{p}=\left(\sum w_{i,light,10\%}\times p_{i,light,10\%}\right)/\left(\sum w_{i,light,10\%}\right)$. Data were subsequently saved in a comma-separated file and processed in statistical analyses.

### 2.5. Statistical Analysis

Only data from the right eye were analyzed and reported. Reproducibility analyses were performed using the method described by Bland and Altman [26,27]. Thus, we assessed the agreement between measurements (i.e., over time and between reading methods) by plotting the difference in measurements for each participant against their average (i.e., Bland–Altman plot). Mean difference between measurements (bias) and limits of agreement (LOA) were calculated, the latter as bias ± 1.96 × standard deviations (SD) of the bias. The assumption that differences followed a normal distribution was tested using quantile–quantile plots (Q–Q plots). Outliers (more than 3 SD) were excluded from the Bland–Altman plots but included in data tables. We used 1.96 × SD of the bias to calculate an agreement coefficient and an inter-method repeatability coefficient when assessing the reproducibility over time and between reading methods, respectively. Reproducibility was higher when the coefficients were lower. Importantly, the observed values of the bias and LOA were only estimates based on the current sample; therefore, 95% confidence intervals (CI) were calculated. The standard error (SE) of the bias and LOA was defined as $\sqrt{SD^{2}/n}$ and $\sqrt{3\times SD^{2}/n}$, respectively, where *n* was the sample size [26]. Using the appropriate value from the *t* distribution with *n* − 1 degrees of freedom, the 95% CI was calculated as the observed estimate ±*t* × SE of the estimate [26]. We used Pearson’s correlation test to evaluate the potential relationship between differences and averages. In case of significant correlation, we calculated the measurements’ ratios and estimated the bias and LOA from these ratios. This approach to removing a relationship between differences and averages was equivalent to a logarithmic transformation because the logarithm of the ratio of two values is identical to the difference between their logarithms [27].

All statistical analyses were performed using R statistical software (version 4.1.0) [28]. The ggplot2 package [29] was used to create the Bland–Altman plots.

## 3. Results

A total of fifty subjects were screened for eligibility at the Copenhagen University Hospital. Of these, 43 participants completed both the screening and baseline visits and were included in the present study. Mean (SD) age of included participants was 9.8 (1.7) years, and 25 (58%) children were girls. Mean (SD) AL was 24.58 (0.87) mm and mean (SD) SE was −2.96 (1.33) D. Median (interquartile range) interval between screening and baseline visit was 15 (23.5) days.

### 3.1. Pupil Diameter

The automated reading showed a consistent trend toward larger pupil sizes, compared to human-assisted readings. This was most pronounced under mesopic conditions, see Table 1. There were two examples of the differences between human-assisted and automated readings which are presented in Figure 1 and Figure 2.

### 3.2. Reproducibility over Time

**Figure 3:**
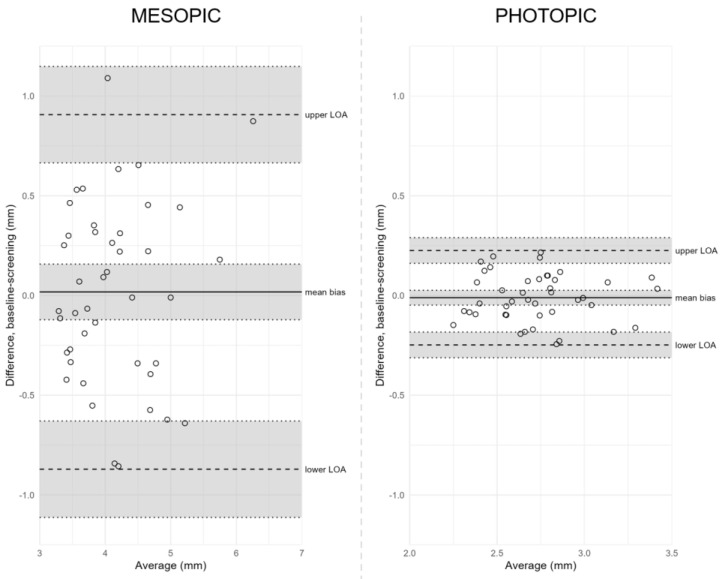
Bland–Altman plots of agreement between visits. **Notes:** Bland–Altman plots of agreement between human-assisted readings of screening and baseline measurements under mesopic (left) and photopic (right) conditions. Differences between measurements are plotted against their averages. The solid line marks the mean difference in pupil size between visits, the dashed lines mark the lower and upper limits of agreement (LOA), and grey shaded areas cover 95% CI of calculated estimates. Please note that the scales in Figure 3 and Figure 4 are different. **Abbreviations:** CI = confidence interval; LOA = limits of agreement.

Reproducibility over time was assessed by comparing pupil size measurements between screening and baseline visits using human-assisted readings (Table 2 and Figure 3). The mean difference between mesopic measurements at screening and baseline was 0.02 mm (95% confidence interval [CI], −0.12 to 0.16), with LOA from −0.87 mm (95% CI, −1.11 to −0.63) to 0.91 mm (95% CI, 0.67 to 1.15). We found no significant correlation between differences and averages (*p* = 0.58).

Under photopic conditions, the mean difference between visits was −0.01 mm (95% CI, −0.05 to 0.03), with LOA from −0.25 mm (95% CI, −0.31 to −0.18) to 0.23 mm (95% CI, 0.16 to 0.29). The differences and averages were not significantly correlated (*p* = 0.82). The agreement coefficients under mesopic and photopic conditions were 0.89 mm and 0.24 mm, respectively, indicating higher reproducibility of photopic measurements when compared over time.

### 3.3. Reproducibility between Human-Assisted and Automated Readings

**Figure 4:**
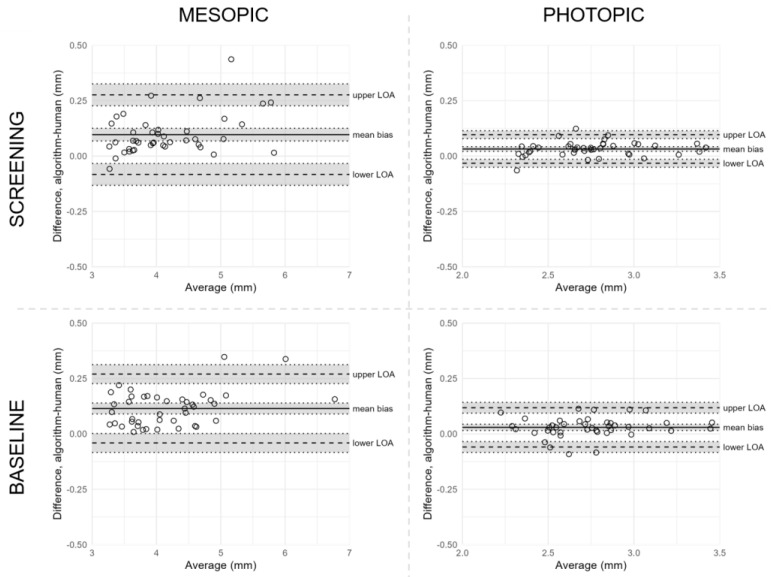
Bland–Altman plots of agreement between reading methods under mesopic and photopic conditions. **Notes:** Bland–Altman plots of agreement between reading methods under mesopic (left) and photopic (right) conditions at screening (upper) and baseline (lower). Differences between human-assisted reading and automated reading are plotted against their averages. The solid line marks the mean difference in pupil size between reading methods, the dashed lines mark the lower and upper limits of agreement (LOA), and grey shaded areas cover 95% CI of calculated estimates. Please note that the scales in Figure 3 and Figure 4 are different. **Abbreviations**: CI = confidence interval; LOA = limits of agreement.

Reproducibility between reading methods was assessed by comparing pupil size measurements captured by human-assisted or automated reading (Table 2 and Figure 4). Under mesopic conditions, we found significant correlations between differences and averages at both screening and baseline visits, indicating greater differences between the reading methods with increasing pupil sizes. Under photopic conditions, the mean difference between human-assisted and automated reading of screening measurements was 0.03 mm (95% CI, 0.02 to 0.04), with LOA from −0.03 mm (95% CI, −0.05 to −0.01) to 0.10 mm (95% CI, 0.08 to 0.11) after exclusion of two outliers. A similar agreement was found between reading methods at the baseline visit, with a mean difference of 0.03 mm (95% CI, 0.02 to 0.04) and LOA from −0.06 mm (95% CI, −0.08 to −0.03) to 0.12 mm (95% CI, 0.09 to 0.14). The inter-method repeatability coefficients indicate that the reproducibility between human-assisted and automated readings was higher under photopic conditions at both visits, although the significant correlations from the mesopic measurements complicate a direct comparison.

### 3.4. Using Ratios to Assess the Reproducibility between Human-Assisted and Automated Readings

**Figure 5:**
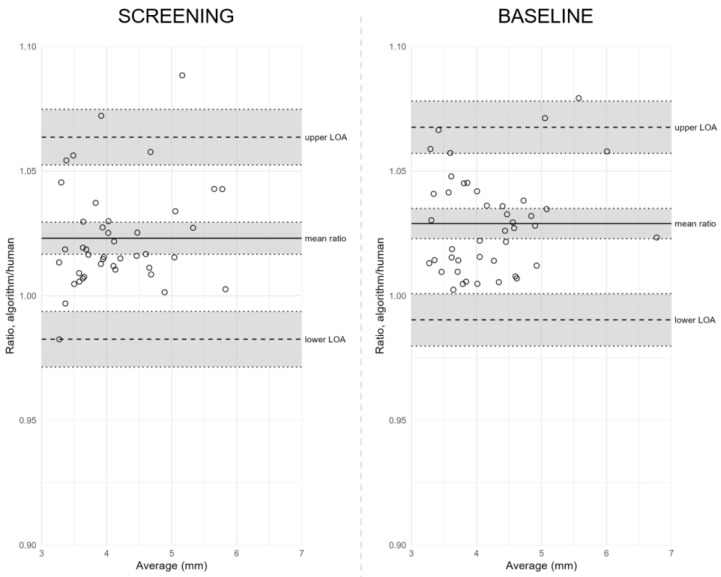
Bland–Altman plots of agreement between reading methods under mesopic conditions using ratios. **Notes:** Bland–Altman plots of agreement between reading methods under mesopic conditions at screening (left) and baseline (right) using ratios. Ratios between human-assisted reading and automated reading are plotted against their averages. The solid line marks the mean ratio of pupil size between human-assisted reading and automated reading, the dashed lines mark the lower and upper limits of agreement (LOA), and grey shaded areas cover 95% CI of calculated estimates. **Abbreviations:** CI = confidence interval; LOA = limits of agreement.

We also assessed the reproducibility between reading methods under mesopic conditions by calculating the measurement ratios and estimated the bias and LOA from these ratios (Table 3 and Figure 5). We did this because the significant correlations observed between the reading method’s differences and averages under mesopic conditions implied that the LOA were too wide for small pupil sizes and too narrow for large pupil sizes. Thus, ratios provided more valid estimates of reproducibility across pupil sizes. At screening, the mean ratio estimate was 1.023 (95% CI, 1.017 to 1.030), with LOA from 0.983 (95% CI, 0.971 to 0.994) to 1.064 (95% CI, 1.052 to 1.075) after exclusion of one outlier. Thus, the automated reading measured an average of 2.3% higher than the human-assisted reading. A similar agreement was present at baseline, with a mean ratio estimate of 1.029 (95% CI, 1.023 to 1.035), with LOA from 0.990 (95% CI, 0.980 to 1.001) to 1.068 (95% CI, 1.057 to 1.078), indicating that the automated reading may measure between 1.0% lower to 6.8% higher than the human-assisted reading for most measurements. We found no significant correlation between ratios and differences at screening or baseline (*p* = 0.19 and *p* = 0.25, respectively).

## 4. Discussion

We investigated two aspects of the reproducibility of pupil size measurements in myopic children using a dedicated pupillometer with human-assisted or automated reading. First, we assessed the reproducibility over time by comparing human-assisted readings of mesopic and photopic measurements from two separate visits. We found that the agreement coefficients under mesopic and photopic conditions were 0.89 mm and 0.24 mm, respectively, indicating that photopic measurements were more reproducible over time. Second, we assessed the reproducibility between human-assisted and automated readings. Again, we found that photopic measurements were more reproducible. Analyses also demonstrated that the reproducibility under mesopic conditions was better described using ratios rather than differences between reading methods. To our knowledge, this study is the first to investigate these aspects of reproducibility in children. Previously, one study assessed the repeatability of pupil size measurements in myopic children [4], whereas other studies examined the repeatability in adults [30,31,32,33].

In contrast to our findings, Tan et al. [4] found that repeatability coefficients were similar under mesopic and photopic conditions, ranging from 0.23 to 0.31 mm. We speculate that mesopic measurements may be highly repeatable when measurements are repeated after 15 min but show less reproducibility when the time interval is increased by days or weeks. Information on long-term reproducibility is essential when pupil size is used to monitor atropine-based myopia progression control. Thus, the difference in agreement coefficients observed in our study may demonstrate that mesopic conditions allow more pupil-regulatory impact from temporal variations in cognitive activity, whereas photopic conditions practically isolate the light stimulus impact on the pupillary response and dissolve the influence of near fixation and cognitive activity. Importantly, we cannot exclude that the observed differences between studies could also be a consequence of using different devices and examination protocols. However, photopic pupil size may be the more relevant clinical measure when pupillometry is used to monitor the side effects of atropine treatment such as photophobia.

In the present study, we used a computer-based pupillometer with continuous binocular pupil size measurements before and during the light stimulus. In addition to the minimum pupil size, dynamic pupillometry holds the potential to assess multiple pupillary parameters, including pupil size before constriction, constriction latency, and constriction velocity. However, in our experience, the built-in reading software was highly susceptible to blink artefacts, and human-assisted manual marking of points of interest was time-consuming. We believe an efficient and objective extraction of these parameters is essential to implement additional pupillary parameters in research and clinical settings, so we developed a customized algorithm for automated data reading.

When comparing human-assisted and automated readings, we observed that the automated reading consistently captured larger pupil sizes, which was most pronounced under mesopic conditions. We believe this bias between reading methods was an inherent consequence of how the customized algorithm behind the automated reading was configured to report a 10% percentile. In practice, the human-assisted reading could report the minimum pupil size based on a single data point, whereas the automated reading depended on the 10% lowest values. This corresponded to approximately 200 milliseconds if all data points were considered valid by the customized algorithm (Figure 1). Human-assisted readings were acquired from the entire 10 s span of light stimulus, whereas automated readings were captured between 2 to 4 s after light onset. It is known that prolonged light stimulus causes the pupil to contract with an initial phasic contraction and a sustained contraction [34]. The pupillary dilation from the phasic to the sustained contraction plateau is termed pupillary escape and is considered an adaptive response to the prolonged light stimuli after the initial phasic pupil contraction [34,35]. Also, it has been shown that the pupillary escape increases as the intensity of the light stimulus decreases [34], which may explain why we observed a greater bias between reading methods under mesopic conditions, especially if the pupillary escape began before the interval of automated reading. The bias between reading methods also included the influence of observed latent pupillary constriction in the late half of the mesopic light stimulus, which was only registered in the human-assisted readings (Figure 2). In hindsight, we do not consider these late constrictions a response to light exposure but rather as observations supporting the idea that mesopic conditions allow more pupil-regulatory impact from near fixation and cognitive activity during measurement. Overall, we think that the customized algorithm behind the automated reading performed within acceptable limits, but the observed biases and inter-method repeatability coefficients demonstrated that reading methods were more reproducible under photopic conditions.

There are some limitations to the current study. Pupillometry was performed in combination with a number of other examinations and on children, which meant that we did not include a dark adaptation period before the measurements were performed. The time interval between screening and baseline visits varied due to the onset of the COVID-19 pandemic but did not exceed four months. However, we cannot rule out seasonal changes in pupil sizes.

Pupillary measurements are performed in clinical trials on myopia management to monitor pupil size changes induced by atropine. These changes must represent actual differences rather than measurement errors or temporal variations in possible confounders such as cognitive activity [4]. When assessing the minimal pupil size as an average of five measurements, our findings suggest that to be considered an actual difference, changes in mesopic and photopic pupil size measurements between two visits must exceed 0.89 mm and 0.24 mm, respectively. Thus, we recommend that researchers in myopia control are cautious when they interpret changes in mesopic pupil size measurements and that they reconsider if mesopic measurements are needed rather than focusing on photopic measurements. Importantly, we advise stakeholders in myopia control to seek consensus on pupillary examinations, including devices, luminosity levels, and examination protocols. In the future, multiple pupillary parameters from automated readings may guide and predict the long-term treatment effect of myopia control.

In conclusion, we used a dedicated pupillometer to investigate two aspects of reproducibility in minimum pupil size measurements. We found that photopic measurements demonstrated the highest reproducibility over time and between reading methods. The reproducibility between reading methods, especially under photopic conditions, also reveals the possibility that automated readings can be used to extract multiple pupillary measurements objectively and time-efficiently. We question whether mesopic measurements are sufficiently reproducible to be of value in clinical trials on myopia control and suggest that photopic measurements may be of greater importance in monitoring the side effects of atropine treatment.

## Figures and Tables

**Table 1:** Mean pupil size measurements.

	Screening		Baseline	
Reading Method	Mesopic	Photopic	Mesopic	Photopic
Human-assisted, mm	4.12 (0.70)	2.73 (0.29)	4.14 (0.74)	2.72 (0.28)
Automated, mm	4.24 (0.76)	2.76 (0.30)	4.26 (0.78)	2.76 (0.29) †

**Notes:** Measurements are presented as mean (SD). **Footnotes:** †, one participant fell below the quality threshold (N = 42).

**Table 2:** Reproducibility of pupil size measurements between visits and reading methods.

		N	Mean Difference (95% CI)	Limits of Agreement (95% CI)		Coefficient	Correlation between Averages and Differences
	**Comparisons**		Bias (mm)	Lower LOA (mm)	Upper LOA (mm)	1.96 × SD (mm)	Pearson’s *r* *p* Value
**MESOPIC**	**Between Visits**						
	Screening vs. Baseline	43	0.02 (−0.12 to 0.16)	−0.87 (−1.11 to −0.63)	0.91 (0.67 to 1.15)	0.89	*r* = −0.088 *p* = 0.58
	**Between Reading Methods**						
	Screening						
	All	43	0.11 (0.07 to 0.15)	−0.14 (−0.21 to −0.07)	0.36 (0.29 to 0.43)	0.25	*r* = 0.48 *p* = 0.0037
	Outlier(s) excluded	42	0.10 (0.07 to 0.13)	−0.08 (−0.13 to −0.03)	0.28 (0.23 to 0.33)	0.18	*r* = 0.39 *p* = 0.011
	Baseline						
	All	43	0.12 (0.09 to 0.15)	−0.06 (−0.11 to −0.01)	0.30 (0.25 to 0.35)	0.18	*r* = 0.45 *p* = 0.0022
	Outlier(s) excluded	42	0.11 (0.09 to 0.14)	−0.04 (−0.08 to 0.00)	0.27 (0.23 to 0.31)	0.15	*r* = 0.38 *p* = 0.014
**PHOTOPIC**	**Between Visits**						
	Screening vs. Baseline	43	−0.01 (−0.05 to 0.03)	−0.25 (−0.31 to −0.18)	0.23 (0.16 to 0.29)	0.24	*r* = −0.035 *p* = 0.82
	**Between Reading Methods**						
	Screening						
	All	43	0.03 (0.01 to 0.05)	−0.09 (−0.12 to −0.05)	0.15 (0.12 to 0.18)	0.12	*r* = 0.27 *p* = 0.079
	Outlier(s) excluded	41	0.03 (0.02 to 0.04)	−0.03 (−0.05 to −0.01)	0.10 (0.08 to 0.11)	0.06	*r* = 0.16 *p* = 0.31
	Baseline	42 †	0.03 (0.02 to 0.04)	−0.06 (−0.08 to −0.03)	0.12 (0.09 to 0.14)	0.09	*r* = 0.11 *p* = 0.47

**Notes:** Reproducibility of mesopic and photopic pupil size measurements between visits and between reading methods. Comparisons over time were performed using human-assisted readings, whereas comparisons between reading methods compared human-assisted readings against automated readings. Bland–Altman plots are shown accordingly in Figure 3 and Figure 4. Mean difference and limits of agreement (LOA) are presented asestimatese (95% CI). Outliers were defined as more than 3 SD. An agreement coefficient and an inter-method repeatability coefficient were calculated when assessing the agreement over time and between reading methods, respectively. Pearson’s correlation test was used to assess potential correlation between differences and averages. If a significant correlation was found, we calculated the measurements’ ratios and estimated the bias and LOA from these ratios, as presented in Table 3 and Figure 5. **Footnotes:** †, one participant fell below the quality threshold (N = 42). **Abbreviations:** CI = confidence interval; LOA = limits of agreement; *r* = Pearson’s correlation coefficient; *p* = *p* value; SD = standard deviation.

**Table 3:** Reproducibility of mesopic pupil size measurements between human-assisted and automated readings using ratios.

		N	Mean Ratio (95% CI)	Limits of Agreement (95% CI)		Coefficient	Correlation Between Ratios and Differences
	**Comparisons**			Lower LOA	Upper LOA	1.96 × SD	Pearson’s *r* *p* Value
**MESOPIC**	**Between Reading Methods**						
	Screening						
	All	43	1.026 (1.017 to 1.034)	0.973 (0.958 to 0.987)	1.079 (1.065 to 1.093)	0.053	*r* = 0.31 *p* = 0.047
	Outlier(s) excluded	42	1.023 (1.017 to 1.030)	0.983 (0.971 to 0.994)	1.064 (1.052 to 1.075)	0.041	*r* = 0.21 *p* = 0.19
	Baseline						
	All	43	1.029 (1.023 to 1.035)	0.990 (0.980 to 1.001)	1.068 (1.057 to 1.078)	0.039	*r* = 0.18 *p* = 0.25

**Notes:** Reproducibility of mesopic pupil size measurements between human-assisted and automated readings at screening and baseline using ratios. Ratios between human-assisted and automated readings were introduced to eliminate the identified correlation between differences and averages in mesopic measurements. Bland–Altman plots are shown accordingly in Figure 5. Mean ratio and limits of agreement are presented as estimates (95% CI). Outliers were defined as more than 3 SD. An inter-method repeatability coefficient was calculated when assessing the agreement between reading methods. Pearson’s correlation test was used to assess the potential relationship between ratios and averages. **Abbreviations:** CI = confidence interval; LOA = limits of agreement; *r* = Pearson’s correlation coefficient; *p* = *p* value; SD = standard deviation.

## Data Availability

The anonymized datasets from the current study are available from the corresponding author on reasonable request.
